# Comparing Farmers’ Market Revenue Trends Before and After the Implementation of a Monetary Incentive for Recipients of Food Assistance

**DOI:** 10.5888/pcd11.130347

**Published:** 2014-05-22

**Authors:** Darcy A. Freedman, Amy Mattison-Faye, Kassandra Alia, M. Aaron Guest, James R. Hébert

**Affiliations:** Author Affiliations: Amy Mattison-Faye, Kassandra Alia, M. Aaron Guest, James R. Hébert, University of South Carolina, Columbia, South Carolina.

## Abstract

**Introduction:**

We examined the influence of an intervention to increase fruit and vegetable purchases at farmers’ markets for recipients of food assistance, Shop N Save (SNS), on revenue trends at a farmers’ market located at a federally qualified health center (FQHC) in rural South Carolina. We compared revenue trends for 20 weeks before the intervention (2011) and 20 weeks after (2012).

**Methods:**

SNS provided one $5 monetary incentive per week to customers spending $5 or more in food assistance at the farmers’ market. SNS was available to any farmers’ market customer using Supplemental Nutrition Assistance Program (SNAP), Special Supplemental Nutrition Program for Women, Infants, and Children (WIC), and/or Senior or WIC Farmers’ Market Nutrition Program (FMNP) vouchers. Sales receipts were recorded for each transaction at the farmers’ market to document payment type and the cost of the purchase. All SNS participants completed a one-time enrollment survey.

**Results:**

A total of 336 customers self-enrolled in SNS from June through October 2012. Most SNS participants were female, African American, and patients at the FQHC. In total, the use of all forms of food assistance (SNAP, WIC, and FMNP) at the farmers’ market increased significantly after the intervention (from 10% before, to 25% after, *P* = .003). Senior FMNP vouchers and SNAP usage increased the most.

**Conclusion:**

Interventions that provide incentives to recipients of food assistance programs at farmers’ markets are a viable strategy for increasing food assistance usage and revenue.

## Introduction

“I know the peaches are worth the price but I just don’t have the money to buy them.”

— Farmers’ market customer, Nashville, Tennessee

Increasing access to farmers’ markets is a proposed strategy for curtailing obesity trends and disparities (1,2) while providing opportunities for community and economic development (3,4). Farmers’ markets have been identified as “keystones” for rebuilding localized food systems (5) and improving fruit and vegetable consumption among Americans (6). The number of farmers’ markets is increasing throughout the United States (7). Communities of color and low-income communities, however, have not benefited equally from the expansion of farmers’ markets (8). In response to evidence suggesting that people with better geographic access to healthy food retailers have healthier diets (9,10), the development of farmers’ markets in communities with limited or no access to food retailers (ie, food deserts) could promote nutrition in low-income, minority communities.

Locating farmers’ markets in food deserts may be an important first step to improving food access among populations disparately burdened by obesity and food insecurity. Yet this step may be insufficient for realizing the health benefits offered by farmers’ markets. As the farmers’ market customer quoted at the beginning of this article explains, economic barriers to food access also exist. People who are concerned about food costs are less likely to shop at farmers’ markets (11). Food costs have been found to be more predictive of obesity than geographic access to healthy food retailers (12). Moreover, addressing economic barriers to food access is particularly important for enhancing the vitality of farmers’ markets in rural contexts (13).

Interventions designed to increase the use of federal food assistance at farmers’ markets are one approach to enhance their economic accessibility. The Supplemental Nutritional Assistance Program (SNAP) is the largest nutrition assistance program in the United States, serving more than 46 million people in 2013. It has been argued that “SNAP represents the greatest untapped potential for farmers’ markets in low-income communities” (14). The number of farmers’ markets accepting SNAP through electronic benefits transfer (EBT) payment systems increased from 18% in 2009 to 21% in 2013 (15,16). During this 5-year time frame (2009-2013), SNAP redemptions at farmers’ markets increased from $4.2 million to $21.1 million (17). Although this trend is promising, 2013 SNAP redemption rates at farmers’ markets (~0.03%) are less than redemption rates in the early 1990s (~0.05%) (15).

Additional federal food assistance programs that can be used at farmers’ markets are the Special Supplemental Nutrition Program for Women, Infants, and Children (WIC) cash value vouchers, and Senior and WIC Farmers’ Market Nutrition Program (FMNP) vouchers. These vouchers have the potential to increase access to produce among low-income seniors and mothers or caregivers and increase economic opportunity for farmers.

Interventions designed to improve access to EBT at farmers’ markets have resulted in improvements in SNAP use among low-income consumers. Providing wireless EBT terminals at farmers’ markets has increased SNAP sales (18,19). Mandating EBT and SNAP access at all farmers’ markets in the city of San Francisco was associated with substantial increases in overall sales at the farmers’ markets (20). These findings reflect the added benefit of integrating economic access initiatives into interventions designed to improve geographic access to healthy foods.

Monetary incentive programs are another approach for increasing economic access to farmers’ markets. These programs typically offer a one-to-one match of food assistance dollars (typically SNAP) used at farmers’ markets with a limit ranging from $5 to $30 in matching funds per shopping day and have resulted in substantial increases (doubling or more) in SNAP usage at participating farmers’ markets (21).

The objective of our study was to examine the influence of an intervention “Shop N Save” (SNS), on farmers’ market revenue. The intervention included a federal monetary incentive to increase fruit and vegetable purchases at farmers markets for food assistance recipients. The study occurred at a farmers’ market based at a federally qualified health center (FQHC) in rural South Carolina.

## Methods

The farmers’ market operates in a predominantly minority (63% African American) rural county at an FQHC in South Carolina. FQHCs are community-driven, nonprofit health care delivery organizations supported by the US Health Resources and Services Administration. More than 1,000 FQHCs serve medically underserved populations and medically underserved areas. The FQHC targeted in this study provides health care to more than 25,000 patients. Most are African Americans with incomes 100% or less of the federal poverty level. This FQHC has high patient volume and a large staff (ie, potential shopping base) and is located near a large subsidized apartment complex, businesses, schools, shopping, medical centers, and a bus stop (22).

**FQHC-based farmers’ market.** A multivendor, produce-only farmers’ market opened at the FQHC in 2011 and currently remains in operation from June through October each year. We compared sales receipts before and after the introduction of SNS to examine changes in revenue trends at the farmers’ market. This analysis focused on the 2011 and 2012 seasons, including a total of 40 market days — 20 before SNS (2011) and 20 after SNS (2012).

The market was organized through a community–university partnership using a community-based participatory research approach (23). It is managed by a farmers’ market manager (a community member) with guidance from a Community Advisory Council. The goals of the market, developed through a community visioning process, are to increase access to fruits and vegetables and improve diet among residents in the rural county and to increase economic opportunity for participating small-scale farmers. The market is open 1 day per week (Fridays) for 4 hours. On average, 5 farmers sell at the market, which is authorized to accept SNAP through a central point-of-purchase EBT system. Most farmers’ market vendors are authorized by the South Carolina Department of Agriculture to accept WIC as well as Senior and WIC FMNP vouchers.

**Shop N Save (SNS).** SNS is a monetary incentive for recipients of food assistance that was implemented during the 2012 farmers’ market season. It was developed in response to customer feedback and sales trends from the 2011 season that suggested the market was not adequately reaching consumers with federal food assistance. SNS is a self-enrollment intervention that provided one $5 monetary incentive per week to farmers’ market customers who spent $5 or more at the market using SNAP, WIC, and/or Senior or WIC FMNP vouchers. Farmers’ market customers could enroll in SNS after they made a purchase of $5 or more with food assistance, which was documented on a sales receipt. All enrollees were assigned a unique identification number to track their market usage over time. This study was reviewed and approved by the Institutional Review Board at the University of South Carolina.

### Data collection

Each sales transaction made at the farmers’ market was recorded manually by trained research assistants. On each receipt, the following information was recorded: date of transaction, customer type (patient, staff, or community member), total cost, and payment type. Additionally, the SNS unique identification number was recorded for all transactions made by a SNS customer. Payment type was recorded as cash or check, SNAP, WIC or WIC FMNP voucher, Senior FMNP voucher, SNS coupon, or other payments (eg, prescription, study incentive, community coupon). WIC and WIC FMNP were recorded together because these coupons were difficult to differentiate. Each sales receipt included one or more sales transactions (ie, methods of payment); thus, the number of sales receipts is less than the total number of sales transactions.

A one-time survey was completed by all participants in SNS when they enrolled in the intervention. The brief 12-item survey was completed individually or with assistance from study staff. Several survey items were based on the Center for Disease Control and Prevention’s (CDC’s) Behavioral Risk Factor Surveillance System (demographics, self-reported health status, stress related to purchasing nutritious foods). Our team developed the remaining items on the basis of prior research.

### Analysis

Descriptive statistics, including frequencies, were used to examine the prevalence of farmers’ market use and payment trends before and after the implementation of SNS. We used χ^2^ tests to assess differences in the frequency of market use; *t* tests and analyses of variance with Bonferroni correction were used to measure differences in revenue trends and payment type using sales receipts from before the SNS intervention and after it. All analyses were conducted using SAS version 9.3 for Windows (SAS Institute Inc, Cary, North Carolina) with statistical significance set at .05.

## Results

A total of 336 people enrolled in SNS from June through October 2012 (Table 1). SNS enrollment decreased linearly over time; the greatest number of participants enrolled in June (37.8%) and the fewest in October (9.2%). Most participants (57.6%) reported they had never been to the farmers’ market before enrolling in SNS. Most participants were women (90.7%), African American (89.9%), and currently patients at the FQHC (53.6%). SNS participants reported high rates of diet-related health impairments, including high blood pressure (50.6%), diabetes (29.5%), and arthritis (29.8%), and 29.2% reported their health status was “fair” or “poor.”

**Table 1:** Demographic Characteristics of Shop N Save (SNS) Participants (N = 336)

Characteristic	Percentage
**Sex**	
Female	90.7
Male	9.3
**Race**	
African American	89.9
White	7.1
Hispanic or Latino	1.8
Native American	0.6
Asian	0.6
**Patient at federally qualified health center **	
Yes	53.6
No	46.4
**Type of household food assistance (check all that apply)**	
Supplemental Nutrition Assistance Program (SNAP)	51.8
Supplemental Nutrition Program for Women, Infants, and Children (WIC)	22.9
WIC Farmers’ Market Nutrition Program	16.7
Senior Farmers’ Market Nutrition Program	51.8
**Total no. of forms of household food assistance**	
One	66.7
Two	23.5
Three	9.8
**Child(ren) in household**	
Yes	38.6
No	61.4
**Barriers to purchasing fresh fruits and vegetables (check all that apply)**	
Lack of transportation	16.4
Stores or markets are too far away	8.6
Cost of food	47.6
My budget	27.7
Don’t like veggies	0.9
Don’t know how to cook veggies	0.6
No challenges	25.3
**Worried about having enough money to buy nutritious meals in past year**	
Never	16.8
Rarely	20.1
Sometimes	43.6
Usually	10.7
Always	11.9
**Self-reported health status**	
Poor	3.1
Fair	26.1
Good	43.9
Very good	16.3
Excellent	10.7
**Self-reported disease status (check all that apply)**	
Diabetes	29.5
High blood pressure	50.6
Arthritis	29.8
Obesity	14.0
Heart disease	9.8
Gallbladder	1.2
Cancer (ever)	3.6
**SNS enrollment month**	
June	37.8
July	21.7
August	19.9
September	11.3
October	9.2

Percentages based on valid percentage.

All SNS participants had 1 or more forms of food assistance: 66.7% had 1 form, 23.5% had 2 forms, and 9.8% had 3; most had SNAP (51.8%) or Senior FMNP vouchers (51.8%) or both. Despite having federal food assistance benefits, two-thirds of the sample reported that over the past year they were sometimes, usually, or always worried about having enough money to buy nutritious meals. At SNS enrollment, participants indicated the most common barriers to purchasing fresh fruits and vegetables were financial (eg, cost of food, budget) and logistical (eg, lack of transportation, stores/markets too far away).

During the 2 seasons (40 weeks), a total of $30,005.33 in revenue was generated for farmers selling at the market. Revenue increased significantly from $14,285.60 before SNS to $15,719.73 after SNS (*P* < .001).

In both seasons, cash was the most common form of payment at the market; however, the percentage of transactions paid in cash decreased significantly after SNS (71% before SNS and 47% after SNS, *P* < .001). The percentage of transactions paid using all forms of food assistance (eg, SNAP, WIC or WIC FMNP, Senior FMNP) increased significantly (10% before SNS and 25% after, *P* = .003). Senior FMNP voucher usage increased the most, from 4% before SNS to 14% after SNS (*P* < .001). The percentage of SNAP transactions increased from 2% to 5% (*P* < .001).

**Figure 1:**
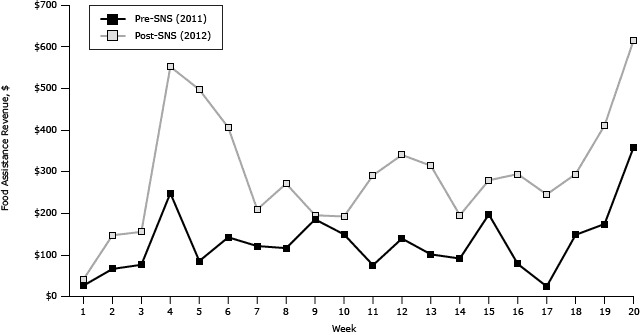
Total food assistance revenue by week, before Shop N Save intervention (2011) and after Shop N Save intervention (2012). Abbreviation: SNS, Shop N Save. **Table:** 

Use of federal food assistance was more consistent at the farmers’ market after SNS. All of the post-SNS market days involved transactions paid with SNAP, WIC or WIC FMNP, and Senior FMNP. Before the SNS intervention, 40% of the weeks did not include transactions paid with SNAP, 10% did not include payments with WIC or WIC FMNP, and 5% did not include payments with Senior FMNP. Food assistance revenue was consistently higher after SNS, with 10% of the pre-SNS market dates achieving $200 or more in food assistance revenue compared with 70% post-SNS (Figure 1).

Week	Total Food Assistance Revenue in 2011 (Pre-SNS), $	Total Food Assistance Revenue in 2012 (Post-SNS), $
1	25	40
2	65	146
3	75	154
4	247	551
5	84	496
6	141	405
7	120	208
8	115	270
9	184	194
10	148	191
11	74	290
12	138	339
13	100	313
14	90	194
15	196	278
16	77	293
17	23	244
18	147	293
19	173	410
20	357	614

Total food assistance revenue more than doubled after the intervention ($2,577.75 vs $5,921.65, *P* < .001) (Table 2). The largest increase in food assistance revenue was related to SNAP ($286.75 vs $1,181.65, *P* = .02) and Senior FMNP ($937.00 vs $3,234.00, *P* = .004). The use of SNS matching coupons at the market accounted for 16% of all sales transactions in 2012, for a total of $3,071 in revenue for participating farmers.

**Table 2:** Farmers’ Market Transactions Before (2011) and After Shop N Save (SNS) (2012) by Payment Type

Type of Payment	Total	Before SNS (2011)		After SNS (2012)		*P* Value
	N ($)	N (%)	Total Purchase/Transaction (Average Purchase/Transaction), $	N (%)	Total Purchase/Transaction (Average Purchase/Transaction), $	
Total transactions^a^	7,357 (30,005.33)	3,667 (100)	14,285.60 (3.90)	3,690 (100)	15,719.73 (4.26)	<.001
Cash	4,346 (14,877.18)	2,595 (71)	8,803.10 (3.39)	1,751 (47)	6,074.08 (3.47)	.39
Shop N Save matching coupon^b^	607 (3,071.00)	NA	NA	607 (16)	3,071.00 (5.06)	NA
Total, all food assistance	1,276 (8,499.40)	359 (10)	2,577.75 (7.18)	917 (25)	5,921.65 (6.46)	.002
SNAP	245 (1,468.40)	58 (2)	286.75 (4.94)	187 (5)	1,181.65 (6.32)	.02
WIC or WIC FMNP	375 (2,860.00)	169 (5)	1,354.00 (8.01)	206 (5)	1,506.00 (7.31)	.08
Senior FMNP	656 (4,171.00)	132 (4)	937.00 (7.10)	524 (14)	3,234.00 (6.17)	.004
Other payment	1,128 (3,557.75)	713 (19)	2,904.75 (4.07)	415 (11)	653.00 (1.57)	<.001

Abbreviations: NA, Not available; SNAP, Supplemental Nutrition Assistance Program; WIC, Supplemental Nutrition Program for Women, Infants, and Children; FMNP, Farmers’ Market Nutrition Program.

a Total transactions includes all payment types recorded on the receipts. One receipt may result in 3 transactions, for instance, if 3 different payment types were used.

b Shop N Save matching coupons were worth $5 each and were provided to SNS participants after they spent ≥$5 in food assistance at the farmers’ market. One coupon could be redeemed per week.

**Figure 2:**
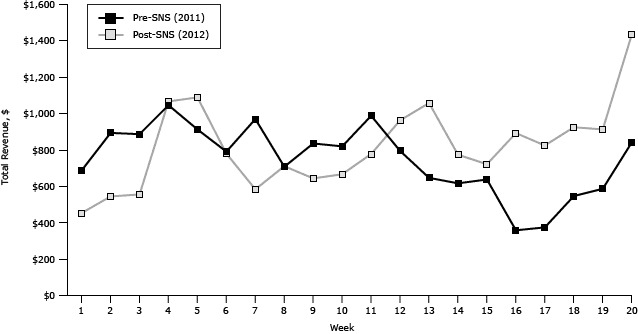
Total revenue by week, before Shop N Save intervention (2011) and after Shop N Save intervention (2012). Abbreviation: SNS, Shop N Save. **Table:** 

Revenue trends at the farmers’ market varied by year. Before SNS, revenue was higher during the beginning of the farmers’ market season (weeks 1–10), yet, during the latter part of the season (weeks 11–20), revenue trends were higher after SNS (Figure 2). Interestingly, the last market day for both seasons resulted in the greatest revenue for that year, followed by the fourth market day. These dates corresponded with the last and first days, respectively, that the FMNP vouchers could be redeemed at the farmers’ market for that season. Overall, in a week-by-week comparison, 60% of the farmers’ market days had higher revenue after SNS than before.

Week	Pre-SNS, 2011, $	Post-SNS, 2012, $
1	655	421
2	861	512
3	854	523
4	1,013	1,034
5	880	1,056
6	758	747
7	937	551
8	675	678
9	803	611
10	787	634
11	955	745
12	762	930
13	614	1,025
14	584	741
15	605	689
16	326	860
17	342	792
18	514	892
19	554	880
20	808	1,400

## Discussion

This is one of the first studies to use objective measures of sales transactions at a farmers’ market to examine the influence of an intervention that offered a monetary incentive to low-income consumers to purchase fruits and vegetables at a farmers’ market. Our findings from rural South Carolina corroborate results from the Health Bucks food assistance matching program in New York City, which emphasized the usefulness of these types of programs for improving affordable access to fruits and vegetables among low-income, urban populations (24). Our data suggest that the food assistance matching intervention was effective not only in increasing food assistance revenue at the farmers’ market but also may have facilitated the farmers’ market’s economic sustainability. Four times more SNAP dollars and 3.5 times more Senior FMNP vouchers were used to purchase produce at the farmers’ market after SNS was implemented. WIC usage remained relatively constant during both farmers’ market seasons, which highlights the importance of additional targeted interventions to increase farmers’ market usage among WIC recipients. We noticed a sharp uptake in food assistance revenue corresponding to the beginning and expiration dates for the FMNP vouchers. The time-sensitivity of the FMNP vouchers may have created a sense of urgency at the beginning but also at the end of the program before they expired. Similar temporal trends were not found for SNAP. Offering farmers’ market incentives with different expiration dates over a season may be one strategy for promoting sustained farmers’ market use among food assistance recipients.

SNS enhanced the economic viability of the rural farmers’ market through increased food assistance revenue, although some improvements in revenue would be expected because of normal growth related to operating a second season. This supports research by Schmit and Gomez (13), who posit that farmers’ markets in rural areas would be more effective if they address the economic constraints among the rural poor. Locating farmers’ markets at FQHCs may further enhance their success by providing a customer base that could be expanded by integrating referrals to the farmers’ markets in preventive health care practice. Future efforts are necessary to facilitate improvements in cash revenue to maximize the viability of farmers’ markets.

Food assistance matching interventions such as SNS represent an approach to health promotion that builds on behavioral economics, which emphasizes the importance of immediate responses to health promotion actions (25). Incentive programs aimed at promoting healthy behaviors may be more effective if they offer “small but tangible and frequent positive feedback or rewards” (23). Providing a small monetary match to food assistance consumers is a tangible reward for making the decision to purchase fruits and vegetables using food assistance dollars. Garnering public support for health behavior incentive programs can be challenging (25,26), but support is more likely if the public believes the incentivized intervention is effective (26).

Monetary incentives designed to promote healthier food purchases and consumption have been found to be “unambiguously” effective (27). A few studies highlight a dose–response effect between the amount of monetary incentive and healthier food purchases and consumption (28,29). A study by Freedman and colleagues (30) found that a farmers’ market intervention combined with a monetary incentive program was effective at improving fruit and vegetable intake among low-income patients with diabetes, and patients with diabetes who exclusively relied on the monetary incentives to purchase produce were more likely to improve fruit and vegetable consumption. The effectiveness of farmers’ market interventions at increasing fruit and vegetable consumption may increase public favor for expanding food assistance incentive programs. Public and political support may be enhanced by highlighting the dual benefit of farmers’ market food assistance incentive programs for both low-income consumers and small-scale farmers. In South Carolina, we presented the results of this study to community advocates and eventually to members of the General Assembly, emphasizing the dual benefits of the food assistance monetary incentive programs for low-income consumers and farmers (31). This framing, supported by our data and the leadership of an antipoverty advocacy organization (Appleseed Legal Justice), resulted in the adoption of a proviso by the South Carolina General Assembly to allocate $1.892 million to support a “double bucks” program in the state that will offer an incentive for SNAP recipients to purchase fruits and vegetables.

This study has both strengths and limitations. Although the pre/post design allows for an examination of revenue trends before and after the SNS matching intervention, our design did not include a comparison site. The SNS program was a self-enrollment program. People who opted to enroll in SNS may be different from those who did not. Although this selection bias does not necessarily influence revenue trends at the farmers’ market, it may influence the generalizability of the food assistance matching intervention as a strategy to increase access to farmers’ markets among food assistance recipients. Finally, manually recording sales receipts at the farmers’ market is a strength of our study. However, there is a chance that some sales receipts were not recorded because of manual errors. Thus, our findings may underestimate overall market usage.

Our research provides support for a monetary incentive intervention as a strategy for increasing food assistance usage and revenue at a farmers’ market in a rural context. Overall food assistance usage at the farmers’ market more than doubled after the introduction of the SNS matching intervention. Sales trends, particularly for time-limited food assistance such as FMNP, showed sharp increases in usage immediately after the vouchers were distributed and before they expired. Findings may guide implementation of future farmers’ market incentive interventions.
